# 813. Characteristics and Outcomes of Hospitalized Patients with Newly Diagnosed Histoplasmosis

**DOI:** 10.1093/ofid/ofad500.858

**Published:** 2023-11-27

**Authors:** Liam Dalton, Marisa H Miceli

**Affiliations:** University of Michigan Medical School, Ann Arbor, Michigan; University of Michigan, Ann Arbor, Michigan

## Abstract

**Background:**

Histoplasmosis (histo), is the most common cause of hospitalization and death among the endemic mycoses in the US. Clinical presentation varies from asymptomatic to severe, disseminated illness indistinguishable from other infections, which may result in misdiagnosis. Physicians must remain vigilant for histo given the potentially devastating consequences of disseminated infection. We sought to determine the characteristics and outcomes of admitted patients (pt) newly diagnosed with histo at our institution.

**Methods:**

We performed a retrospective evaluation of hospitalized adult pt newly diagnosed with histo via culture, histopathology, or antigen testing (Ag) at the University of Michigan from 1/1/2015-12/31/2022. Demographic, clinical, treatment, & outcome data were collected. We compared the characteristics and outcomes of immunocompromised (IC) pt with those of non-immunocompromised (NIC) pt.

**Results:**

62 pt were included, 51 IC (76%) & 16 NIC (24%). The IC group included pt with solid organ transplants (19), autoimmune disorders treated with immunosuppressive drugs (22), hematologic (4) or solid malignancies & AIDS (1 each). Mean age of IC was 46.13+17.4 yr vs 58.1+16.4 yr among NIC, p=0.02; 74% IC were men vs 80% NIC. Mean BMI in IC pt was 26.8+6.2 vs 24.1+5.2 among NIC, p=0.13. Disseminated histo was more common in IC pt (74.5% vs 46.7%, p=0.048). ICU admission occurred in 46.8% IC vs 40% NIC, p=0.75. All-cause 6-month (m) mortality was 24%: 25.5% IC vs 20% NIC; 3 pt died before diagnosis was made (2 IC; 1 NIC). The initial treatment for most IC pt was amphotericin B (76.6%, p< 0.01). Fifty-two pt were diagnosed using Ag testing (40 IC; 12 NIC); Ag titers above the limit of quantification occurred in 15 IC pt; 14 had disseminated disease. Histo was diagnosed year-round in our pt cohort, but the most pt presented in winter (39%; p=0.02). December was the m with the most cases (27.4%, p< 0.001). There was no statistical difference in diagnostic seasonality between hospitalized IC & NIC pt.

**Conclusion:**

Hospitalized IC pt diagnosed with histo were younger and more frequently had disseminated disease than NIC pt, but morbidity and mortality did not differ. Unlike other reports, at our institution, the most pt with histo were diagnosed during the winter months.

**Disclosures:**

**Marisa H. Miceli, MD**, Astellas: Advisor/Consultant|F2G: Grant/Research Support|Scynexis: Advisor/Consultant|Scynexis: Grant/Research Support

